# Cilnidipine lowered psychological stress-induced increase in blood pressure in a hypertensive man: a case report

**DOI:** 10.1186/1751-0759-1-16

**Published:** 2007-09-27

**Authors:** Sota Hayashida, Takakazu Oka, Sadatoshi Tsuji

**Affiliations:** 1 Psychosomatic Medicine Division, Department of Neurology, University of Occupational and Environmental Health, Japan Iseigaoka 1-1, Yahatanishi-ku, Kitakyushu, 807-8555, Japan

## Abstract

**Background:**

In some hypertensive patients, psychological stress makes blood pressure difficult to control and causes physical symptoms such as headache or dizziness. We report the case of a hypertensive man whose psychological stress-induced increase in blood pressure was attenuated by cilnidipine.

**Case Presentation:**

The patient (a 72-year-old man) had hypertension and was on antihypertensive therapy. When mentally concentrating, he experienced occipital headaches and dizziness, and despite thorough testing, no abnormality was found. He was subsequently referred to our department. The mirror drawing test (MDT), a psychological stress test, increased blood pressure by about 40 mmHg, and the patient described occipital headache. Plasma noradrenaline level also increased from 212 to 548 pg/ml. We therefore switched the patient from nifedipine, an L-type calcium (Ca) channel blocker, to cilnidipine, an L-type/N-type Ca channel blocker with suppressive effects on sympathetic activity. Cilnipidine attenuated MDT-induced an increase in blood pressure and plasma noradrenaline level and prevented the development of headache during testing.

**Conclusion:**

These findings suggest that cilnidipine is a useful antihypertensive agent for hypertensive patients in whom psychological stress causes marked fluctuations in blood pressure.

## Background

In hypertensive patients (especially men), the sympathetic nervous system is overreactive and stress-induced increases in blood pressure are greater in hypertensive than healthy subjects [1-3]. Therefore, in some hypertensive patients, psychological stress makes blood pressure difficult to control, decreasing quality of life (QOL). Furthermore, psychological stress-induced increase in blood pressure has been demonstrated to be an independent contributor to left ventricular hypertrophy in hypertensive men [3,4]. Therefore, control of psychological stress-induced, exaggerated increase in blood pressure is important from the viewpoint of preventing left ventricular hypertrophy as well as improving QOL in hypertensive men.

Cilnidipine (Atelec; Mochida Pharmaceutical, Tokyo, Japan) is a dihydropyridine calcium (Ca) antagonist, which like conventional Ca antagonists exhibits antihypertensive activities *via *L-type Ca channel inhibition, but it also suppresses the sympathetic nervous system *via *N-type voltage-dependent Ca channels [5]. Thus, cilnidipine was demonstrated to suppress stress-induced hypertension in rats [6]. We, therefore, expected that cilnidipine would benefit hypertensive patients with impaired QOL in whom psychological stress alters blood pressure.

We present herein the case of a hypertensive man successfully treated for psychological stress-induced, marked blood pressure increases and occipital headaches that hindered his activities of daily living. The efficacy of cilnidipine was demonstrated using the mirror drawing test (MDT).

## Case Presentation

### Patient

A 74-year-old man.

### Chief complaint

Occipital headache when concentrating.

### Family history

No family history of hypertension.

### Medical history

Nothing of note.

### Life history

No history of smoking. About 350 ml/day of beer.

### History of present illness

The patient was diagnosed with hypertension at about 40 years of age, and he has since been taking slow-release nifedipine tablets. Blood pressure was controlled at about 130/80 mmHg, but in April 200X, he began to experience non-pulsatile occipital headaches and dizziness when concentrating on work or whenever mental focus was required. These conditions were severe enough to impair activities of daily living. For example, on several occasions he had to excuse himself from meetings. Occipital headaches had no relation to nifedipine administration. Also, when the patient experienced occipital headaches, systolic blood pressure was about 50 mmHg higher than usual. His family physician ordered blood tests and head magnetic resonance imaging (MRI), but no abnormalities were found. Since the involvement of psychological stress was suspected, the patient was referred to our department at the end of May and was admitted for thorough testing on June 5.

### Physical findings

Height 161.6 cm, body weight 59.7 kg, body temperature 36.5°C, blood pressure 120/64 mmHg, heart rate (HR) 60 beats/min and regular. No yellowing of the bulbar conjunctiva. No anemia using palpebral conjunctiva hue. No abnormalities in the chest, heart, or respiratory sounds. No abdominal abnormality. No leg edema. No neurological abnormality. Marked bilateral palmar sweating.

### Psychosocial background

The patient was very detail-oriented and a perfectionist. For several years, he had served as a board member of a company as well as a few community and elderly groups. In April, he was particularly busy and under great stress due to personal relationship issues involving some group members.

### Test findings on admission

No abnormal blood count findings. Blood biochemical analysis showed no abnormalities, including total cholesterol (205 mg/dl) and triglycerides (128 mg/dl). Endocrinological testing conducted at rest in the early morning showed no abnormalities: plasma rennin activity, 1.8 ng/ml/hr; aldosterone, 81.0 pg/ml; adrenaline, 27 pg/ml; and noradrenaline (NA) 162 pg/ml. There was no cardiac dilatation (by chest radiography), no adrenal abnormalities (by abdominal ultrasonography [US]), no signs of carotid artery constriction (by US), no abnormality in the skull (by head MRI), and no stenosis (by MRA). The head-up tilt test did not confirm orthostatic hypotension.

### Post-admission course (Fig. 1)

Even after admission, the patient experienced non-pulsatile pain accompanied by heaviness in the back of the head whenever thinking or frustrated. Before admission, the patient was taking four antihypertensive agents (valsartan [80 mg], slow-release nifedipine [40 mg], temocapril [2 mg], and trichloromethiazide [2 mg]), but after admission, use of trichloromethiazide and temocapril was discontinued in that order. To investigate the effects of psychological stress on the patient, the first MDT was performed to measure changes in blood pressure, HR, and plasma NA (the MDT protocol is described below). At 2 min after starting MDT, systolic blood pressure (SBP) and diastolic blood pressure (DBP) increased by up to 38 and 36 mmHg, respectively, before gradually decreasing. In addition, occipital headache was experienced at 2 min after the start of MDT and persisted for 15 min (Fig. 2). On June 21, use of slow-release nifedipine was discontinued and 10 mg of cilnidipine was started. At 1 week after the start of cilnidipine therapy, a second MDT was performed, and the maximum increase in SBP was limited to 27 mmHg. The patient did not experience further bouts of occipital headache. Cilnidipine dosage was increased to 20 mg, and a third MDT showed that the maximum increase in SBP was 21 mmHg. Furthermore, maximum increase in HR during MDT while the patient was on nifedipine therapy was 48 beats/min, and this increase was also suppressed by cilnidipine (at both 10- and 20-mg doses).

**Figure 1:**
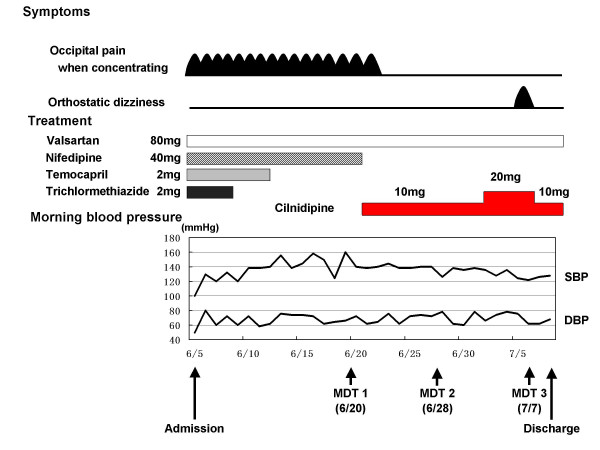
Post-admission clinical course. Doses indicate daily doses. Resting blood pressure was measured while the patient was in a recumbent position at 07:00. MDT: mirror drawing test. MDT1: First MDT on June 20; MDT2: second MDT on June 28; MDT3: third MDT on July 7; SBP: systolic blood pressure; DBP: diastolic blood pressure.

**Figure 2:**
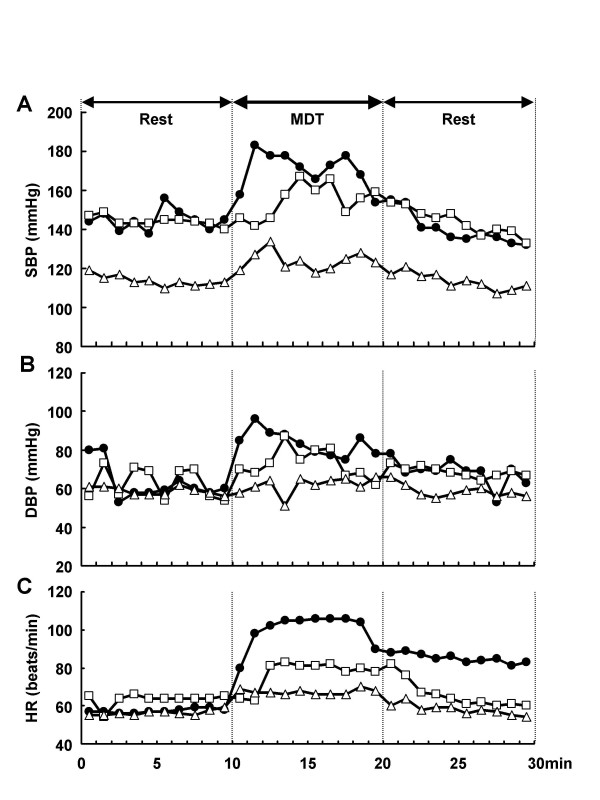
Changes in systolic blood pressure (SBP) (A), diastolic blood pressure (DBP) (B) and heart rate (HR) (C) before and after mirror drawing test (MDT). The MDT was conducted for 10 min from min 10 to min 20. Closed circle: First MDT (40-mg slow-release nifedipine). Open square: Second MDT (10-mg cilnidipine). Open triangle: Third MDT (20-mg cilnidipine). The patient experienced occipital headache 2–15 min after the start of the test during first MDT.

**Figure 3:**
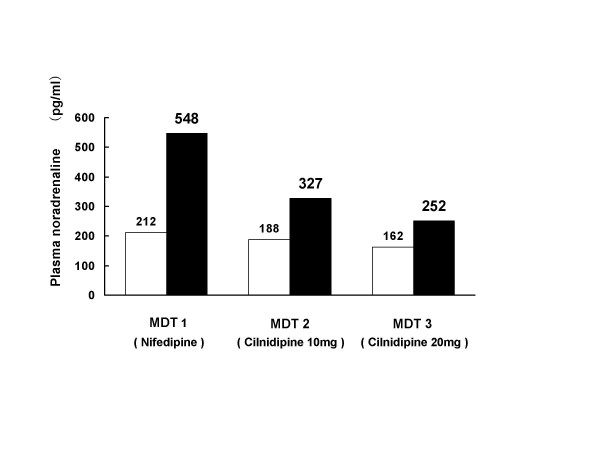
Changes in plasma noradrenaline levels before and after the MDT. Open square: Before MDT. Closed square: Immediately after MDT.

To investigate the effects of psychological stress on sympathetic nerve activities, the level of plasma NA was measured before and immediately after MDT (Fig. 3). Plasma NA increased immediately after MDT by 336 pg/ml while the patient was on nifedipine therapy, but the increase was smaller when the patient was on 10 or 20 mg of cilnidipine (139 and 90 pg/ml, respectively). Cilnidipine thus suppressed blood pressure, HR, and sympathetic activity during the MDT.

However, several days after increasing the cilnidipine dosage to 20 mg, the patient experienced a different type of dizziness when standing up to go to the bathroom. Another head-up tilt test was conducted, and at 1 min after standing up, the patient had a 24 mmHg decrease in SBP. As cilnidipine was considered to have suppressed the sympathetic activation associated with standing, the cilnidipine dosage was reduced to 10 mg. A subsequent head-up tilt test revealed no orthostatic hypotension, and the patient no longer experienced dizziness on standing. While in the hospital, the patient's stress coping skills were evaluated. Although excessive workload was thought to be the cause of his psychological stress, the patient could not abruptly resign from his various posts. He was thus instructed to lighten his workload as much as possible and was discharged on July 11. Since then, he has been free of occipital headaches, and his clinical course has been favorable.

### MDT

At 1 h before start of the test, a line was placed in the right forearm to collect venous blood samples. After resting for ≥ 30 min, the MDT was performed for 10 min. After the end of the MDT, the patient was asked to rest for 10 min. During this time, blood pressure and HR were measured every min. Plasma NA level was measured immediately before and immediately after MDT. MDT was performed three times during hospitalization, and all three tests were administered at about 11:00 A.M. by the same investigator. To avoid habituation to the test, the patient was instructed to start drawing from different positions in different directions and each test was conducted at least seven days apart. Self-reported introspection was recorded following each test and these introspections showed that the degree of frustration experienced during the MDT was comparable each time. Furthermore, during all three tests, the patient was medicated with valsartan. Nifedipine (40 mg) had been discontinued after the first MDT (after breakfast). Cilnidipine administration (10 mg, after breakfast) was initiated on June 21, and the second MDT was performed on June 28. Starting on July 1, cilnidipine dosage was increased to 20 mg (after breakfast), and the third MDT was performed on July 7.

## Discussion

The present study demonstrated that cilnidipine suppressed the MDT-induced increase in blood pressure, tachycardia, and NA in a dose-dependent manner in a hypertensive man.

The sympathetic nervous system of hypertensive animals is overreactive to psychological stress. For example, when subjecting rats to emotional stress (shaking), sympathetic activity and blood pressure increased, but the degree of increase was greater for spontaneous hypertensive rats compared to normotensive rats [7]. Unlike nifedipine, which has no effect on stress-induced hypertension and tachycardia, cilnidipine suppresses these events [6]. Human studies have shown that cilnidipine is more effective than nifedipine or amlodipine and significantly attenuates the white coat phenomenon in hypertensive patients [2], increase in catecholamine levels on the cold pressor test [5], and workday hypertension [8]. The results of the MDT in the present case are well in agreement with these findings.

While our patient was on nifedipine, his SBP, DBP, and HR during MDT increased 38 mmHg, 36 mmHg, and 48 beats/min, respectively. By comparison, mean increases in SBP, DBP, and PR for 10 normotensive subjects at our facility were 12.7 mmHg, 10.5 mmHg, and 7.7 beats/min, respectively. Thus, the sympathetic nervous system of the present patient clearly overreacted to psychological stress. As with conventional Ca antagonists, cilnidipine blocks the Ca^++ ^influx from L-type Ca channels to suppress vasoconstriction, and at the same time suppresses excessive release of NA from sympathetic nerve endings by hindering Ca^++ ^current *via *N-type Ca channels. The latter may account for the inhibitory effect of cilnidipine on stress-induced sympathetic hyperactivity in the present patient.

Furthermore, cilnidipine relieved headaches experienced during the MDT or activities of daily living in this patient. Among hypertensive patients, QOL may be impaired by physical symptoms accompanying hypertension, such as headache and dizziness. Cilnidipine may thus also make hypertensive patients less vulnerable to stress-induced physical complaints. As psychological stress-induced increase in blood pressure has been demonstrated to correlate positively with the left ventricular hypertrophy in hypertensive men [3,4], the inhibitory effect of cilnidipine on the stress-induced increase in blood pressure may also slow the progress of, or prevent, left ventricular hypertrophy in hypertensive patients.

However, when cilnidipine dosage was increased to 20 mg, the patient experienced an orthostatic blood pressure decrease. High-dose cilnidipine may suppress not only psychological stress-induced sympathetic hyperactivity, but also sympathetic reflexes occurring on standing. Caution must be exercised when administering high-dose cilnidipine.

There are several limitations in this study. Firstly, one criticism might be that the results of the 2^nd ^and the 3^rd ^MDT are due to the habituation effect. As was described in the MDT section, we tried to avoid habituation to the test and the patient reported that the degree of frustration felt after each MDT was the same. Therefore, although the habituation effect cannot be totally excluded, it can be minimized. Secondly, it is possible that the following factors accompanying hospitalization affect the results, i.e., dietary changes especially Na intake, relief from daily stress, or reduced anxiety. As the patient was not overweight and his blood cholesterol level was within the normal range, he was served only a low Na diet during hospitalization. However, as the patient was well educated about Na intake, his intake of Na was the same before and after admission. The patient complained of headache even after hospitalization, for example, during his interview. These symptoms decreased after starting cilnidipine and the patient did not experience headache after discharge. Therefore, the involvement of these factors may also be minimum. A placebo effect may be excluded because the patient had an orthostatic decrease in blood pressure at the higher dose of cilnidipine, which could only be due to the pharmacologic effect of cilnidipine.

## Conclusion

These results suggest that cilnidipine is a useful antihypertensive agent in hypertensive patients experiencing marked increases in blood pressure following psychological stress. Cilnidipine may also improve stress-induced physical symptoms in hypertensive patients. However, further study is necessary to generalize the conclusions drawn from this case report.

## Abbreviations

MDT, mirror drawing test; Ca, calcium; QOL, quality of life; MRI, magnetic resonance imaging; HR, heart rate; NA, noradrenaline; US, ultrasonography; SBP, systolic blood pressure; DBP, diastolic blood pressure.

## Competing interests

The author(s) declare that they have no competing interests.
